# Inhibition of NADPH Oxidase Activation by Apocynin Rescues Seizure-Induced Reduction of Adult Hippocampal Neurogenesis

**DOI:** 10.3390/ijms19103087

**Published:** 2018-10-09

**Authors:** Song Hee Lee, Bo Young Choi, A Ra Kho, Jeong Hyun Jeong, Dae Ki Hong, Dong Hyeon Kang, Beom Seok Kang, Hong Ki Song, Hui Chul Choi, Sang Won Suh

**Affiliations:** 1 Department of Physiology, College of Medicine, Hallym University, Chuncheon 24252, Korea; sshlee@hallym.ac.kr (S.H.L.); bychoi@hallym.ac.kr (B.Y.C.); rnlduadkfk136@hallym.ac.kr (A.R.K.); jd1422@hanmail.net (J.H.J.); zxnm01220@gmail.com (D.K.H.); ehdgus2728@naver.com (D.H.K.); ttiger1993@gmail.com (B.S.K.); 2 College of Medicine, Neurology, Hallym University, Chuncheon 24252, Korea; 3 Hallym Institute of Epilepsy Research, Chuncheon 24252, Korea

**Keywords:** epilepsy, pilocarpine, neuron death, NADPH-oxidase inhibitor, oxidative stress, neurogenesis

## Abstract

Apocynin, also known as acetovanillone, is a natural organic compound structurally related to vanillin. Apocynin is known to be an inhibitor of NADPH (Nicotinamide adenine dinucleotide phosphate) oxidase activity and is highly effective in suppressing the production of superoxide. The neuroprotective effects of apocynin have been investigated in numerous brain injury settings, such as stroke, traumatic brain injury (TBI), and epilepsy. Our lab has demonstrated that TBI or seizure-induced oxidative injury and neuronal death were reduced by apocynin treatment. Several studies have also demonstrated that neuroblast production is transiently increased in the hippocampus after seizures. Here, we provide evidence confirming the hypothesis that long-term treatment with apocynin may enhance newly generated hippocampal neuronal survival by reduction of superoxide production after seizures. A seizure was induced by pilocarpine [(25 mg/kg intraperitoneal (i.p.)] injection. Apocynin was continuously injected for 4 weeks after seizures (once per day) into the intraperitoneal space. We evaluated neuronal nuclear antigen (NeuN), bromodeoxyuridine (BrdU), and doublecortin (DCX) immunostaining to determine whether treatment with apocynin increased neuronal survival and neurogenesis in the hippocampus after seizures. The present study indicates that long-term treatment of apocynin increased the number of NeuN^+^ and DCX^+^ cells in the hippocampus after seizures. Therefore, this study suggests that apocynin treatment increased neuronal survival and neuroblast production by reduction of hippocampal oxidative injury after seizures.

## 1. Introduction

An epileptic seizure is a transient or recurrent display of related symptoms due to abnormal excessive or synchronous neuronal activity in the brain. Epilepsy is now termed a disease, rather than a disorder. When epilepsy occurs, there is a high probability that it may develop into recurring epilepsy (chronic epilepsy) [1,2,3]. Epilepsy leads to neuronal death in the hippocampus, which manifests as cognitive and behavioral impairments. Following periods of severe epileptic seizures, there is a dramatic increase in hippocampal cellular proliferation and neurogenesis. Neurogenesis is the process by which neurons are created from neural stem cells, and occurs continuously in certain parts of the adult brain, especially in the subgranular zone (SGZ) of the hippocampal dentate gyrus (DG). Most of the newly born neurons are produced in the SGZ and die shortly after they are born. A smaller fraction are functionally integrated with surrounding brain areas [4]. Neurogenesis is highly influenced by learning and memory, emotions, stress, and other conditions [5]. Neurogenesis in the DG is known to be associated with hippocampal function for learning and memory [6,7]. In particular, neurogenesis occurs in response to brain damage, and studies on neurogenesis have been extensively conducted in various animal models. However, the topic is not without controversy and for decades the effect of seizures on neuron death and neurogenesis has been debated [8,9,10,11].

Epileptic seizure is known to increase hippocampal neurogenesis [12,13]. However, recurrent and continuous seizures can lead to an ongoing increase in oxidative stress and a constant suppression of the hippocampal neurogenic process [14,15,16]. In addition, the reduction of neural development in the hippocampus is one of the primary causes of hippocampus-dependent learning and cognitive memory impairment observed after hippocampal damage due to epilepsy or other diseases [15]. After seizures, oxidative stress is increased, and the perturbation of redox balance in the hippocampus can reduce neurogenesis [14].

Apocynin is structurally related to natural vanillin, which is extracted from various plants and has been studied for its various pharmacological properties [17,18]. Apocynin is also known to inhibit NADPH (Nicotinamide adenine dinucleotide phosphate) oxidase by inhibiting p47phox subunit translocation from the cytoplasm to the plasma membrane area [19,20]. In many other studies, apocynin has been shown to successfully reduce reactive oxygen species (ROS) and reduce cell death [21,22,23]. Our previous studies demonstrated that pilocarpine-induced seizures increased the generation of ROS in hippocampal neurons. Furthermore, treatment with apocynin after seizures reduced neurodegeneration in other studies [24,25]. Our previous study also demonstrated that production of ROS by NADPH oxidase activation induced neuronal cell death after traumatic brain injury or hypoglycemia [21,22,26,27,28]. Although we have demonstrated that effects of NADPH oxidase activation in neuronal death after hypoglycemia [26], traumatic brain injury [22], and epilepsy [21], the efficacy of inhibition of NADPH oxidase activation by apocynin treatment in seizure-induced neurogenesis has not been demonstrated. We hypothesized that, if ROS are constantly produced after a seizure, the rate of newly proliferated neuronal survival is reduced as a function of the ROS production [29,30,31]. In the present study, we found that the survival rate and neurogenesis of hippocampal cells after seizure induction was increased via inhibition of NADPH oxidase. We found that neurogenesis is increased in the early stages of a seizure, but declines after 4 weeks. However, in rats treated with apocynin for 4 weeks after seizures, neuronal death was reduced, and the survival rate of early proliferated cells increased.

Thus, the present study suggests that treatment with apocynin may have a high therapeutic potential for increasing neurogenesis, as well as reducing seizure-induced neuronal death through reduction of ROS production.

## 2. Results

### 2.1. Apocynin Treatment Reduces Seizure-Induced Hippocampal Neuronal Death

**Figure 1:**
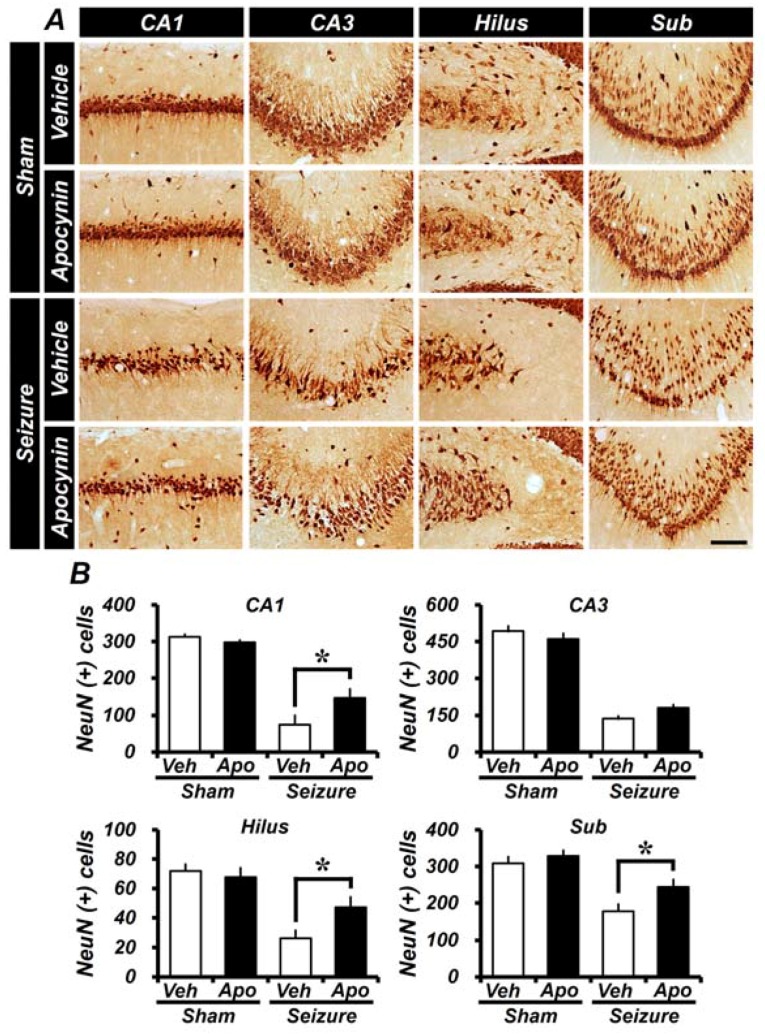
Treatment with apocynin for a week reduces seizure-induced neuronal cell death. Anti-neuron nuclear antigen (NeuN) staining was performed to identify live neurons. (**A**) A micrograph showing live neurons in the sham group, the seizure control group, and the apocynin treatment group. Apocynin treatment shows a substantially increased number of NeuN^+^ cells in the hippocampus of the seizure group. Scale bar = 100 μm. (**B**) The graph shows the average number of live neurons (NeuN^+^ cells) in the hippocampus after seizure compared to the apocynin treatment group. Data: mean ± standard error of the mean (S.E.M.), *n* = 5–6 from each group. * *p* < 0.05. CA3 is not statistically significant. Veh = Vehicle; Apo = Apocynin; CA1 and CA3 = Cornu Ammonis 1 and 3 of the hippocampus area; Sub = Subiculum.

To determine whether apocynin therapy is effective in neuroprotection, we measured the number of live neurons by staining with anti-neuron nuclear antigen (NeuN) one week after the seizure. The number of NeuN^+^ cells was decreased in the Cornu Ammonis 1 and 3 (CA1 and CA3), hilus, and subiculum (Sub) regions of the hippocampus after the seizure (Figure 1A). In the CA1, CA3, hilus, and subiculum areas, the number of NeuN^+^ neurons in the seizure-vehicle-treated group decreased, when compared to the sham-vehicle group. Apocynin treatment for 1 week significantly reduced neuronal death in each region compared to the vehicle-treated seizure group. In the CA3 region, neuronal death was less in the seizure-apocynin group, but this was not significantly different after analysis. This result shows that the number of viable neurons is higher in the apocynin-treated group and that apocynin-treatment reduces seizure-induced neuronal death.

### 2.2. Apocynin Treatment for One Week Reduces Oxidative Damage after Seizure in the Hippocampus

**Figure 2:**
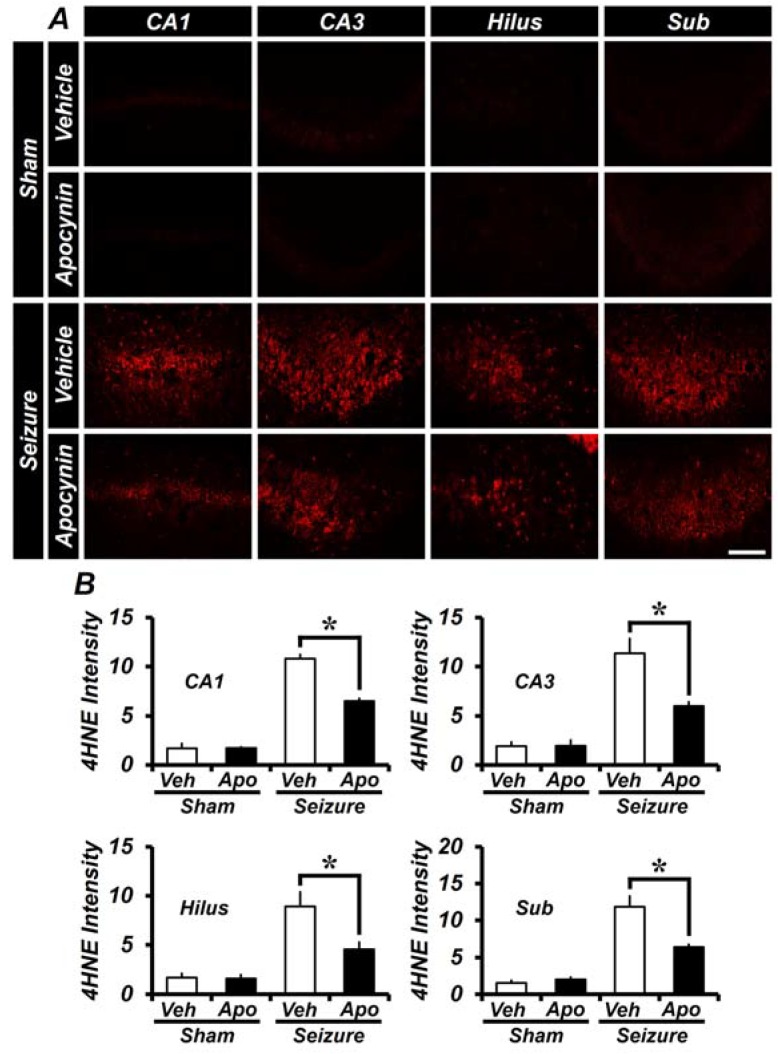
Apocynin administration reduces oxidative injury after seizure. Oxidative stress was observed by anti-4-hydroxynonenal (4HNE) immunostaining after one week of seizure. (**A**) The intensity of 4HNE was increased in the seizure-vehicle group when compared to the sham group. In the seizure-apocynin treated group, the 4HNE intensity was lower than the vehicle treated group. Scale bar = 100 μm. (**B**) The bar graph shows the 4HNE fluorescence intensity of the hippocampus shown in (**A**). Data: Mean ± S.E.M., *n* = 5–6 from each group, * *p* < 0.05.

To assess the degree of oxidative damage in the hippocampus, apocynin was administered for one week and then 4HNE (4-hydroxy-2-nonenal) antibody was used for immunohistochemical staining. In the sham group, there was almost no 4HNE fluorescence staining in both saline injected vehicle and in apocynin injected group. However, in the hippocampus of the pilocarpine-induced seizure group, the intensity of 4HNE fluorescence was increased when compared to sham group. As quantified in Figure 2B, the intensity of 4HNE fluorescence intensity was increased in the hippocampal CA1, CA3, hilus and subiculum compared to the sham group. The 4HNE fluorescence intensity was lower in the apocynin group than in the vehicle group. The intensity of the 4HNE signal in CA1, CA3, hilus and subiculum was 40 ± 2.6%, 47 ± 3.5%, 48 ± 7.5% and 46 ± 3.6% lower, in the apocynin treated group compared to the seizure-vehicle group (Figure 2).

### 2.3. Apocynin Treatment for One Week Does Not Affect the Generation of Progenitor Cells and Production of Neuroblast in the Hippocampus after Seizure

**Figure 3:**
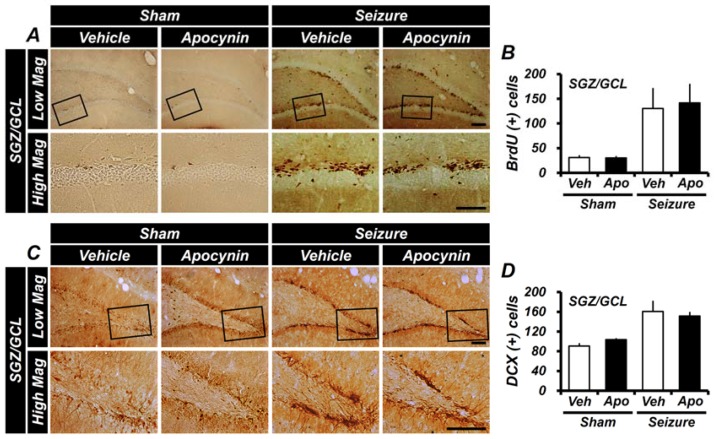
Treatment with apocynin for one week does not affect cell proliferation and neuroblast production after seizures. (**A**) Representative immunohistochemical staining with 5-bromo-2′-deoxyuridine (BrdU) in the subgranular zone (SGZ) of dentate gyrus (DG). A cluster of proliferation cells labeled with BrdU in the SGZ immediately following a seizure is shown. BrdU^+^ cells are increased in the SGZ of the DG both in the vehicle group and in the apocynin group after 1 week of seizures. Boxed areas are higher magnification (bottom row). Scale bar = 100 μm. (**B**) The bar graph shows the quantification of BrdU^+^ cells from the SGZ of the DG. The seizure group, compared with the sham group, showed a greater increase in BrdU^+^ cells. There was no significant difference between the seizure-vehicle- and apocynin-treated groups. (**C**) This figure shows the doublecortin (DCX)-immunoreactive cells in the SGZ of the DG and shows the neuroblast production by enlarging the DG and box area. In the DG of the hippocampal area where a seizure was induced by pilocarpine, more neuroblasts were observed than the uninjured hippocampus. Boxed areas are higher magnification (bottom row). Scale bar = 100 μm. (**D**) The bar graph is the result of counting DCX^+^ cells stained in the DG of the hippocampus. DCX^+^ cells increased more in seizure-induced rats compared with the sham group. There was no significant difference between the vehicle- and apocynin-treated groups. Data: Mean ± S.E.M., *n* = 5–6 from each group. Low Mag, low magnification; High Mag, high magnification.

Immunohistochemical staining with 5-bromo-2′-deoxyuridine (BrdU) and doublecortin (DCX) antibody was performed to determine whether immediate 1-week apocynin treatment affected the production of cellular proliferation and recognizes neuroblasts or immature neurons after a seizure. BrdU was administered twice daily (30 mg/kg, i.p.) to rats 4 days before sacrifice following a pilocarpine-induced seizure. We quantified BrdU^+^ cells showing proliferating cells after a seizure. After one week of pilocarpine-induced seizures, the number of BrdU^+^ cells were significantly increased in the subgranular zone (SGZ) of the vehicle-treated and apocynin-treated groups, respectively, compared to the sham-vehicle-treated group. However, the number of BrdU^+^ cells in the apocynin-treated group was similar between the two groups when compared to the vehicle-treated group after one week of seizures. DCX immunostaining is effective in identifying and quantifying newly born neuroblasts in the adult DG. Detection of immunologic markers using DCX antibodies showed neuroblasts in the SGZ in the DG region. One-week treatment with apocynin increased neuroblasts in both seizure groups compared to the sham-vehicle-treatment group, but did not show any difference in neuroblastogenesis in the SGZ of the DG when compared with seizure-vehicle-treated and apocynin-treated groups. These results indicate that treatment with apocynin for one week after seizure has no effect on seizure-induced cellular proliferation and production of neuroblasts after seizure (Figure 3).

### 2.4. Seizure-Induced Hippocampal Neuronal Death Is Reduced by Apocynin Treatment for Four Weeks

**Figure 4:**
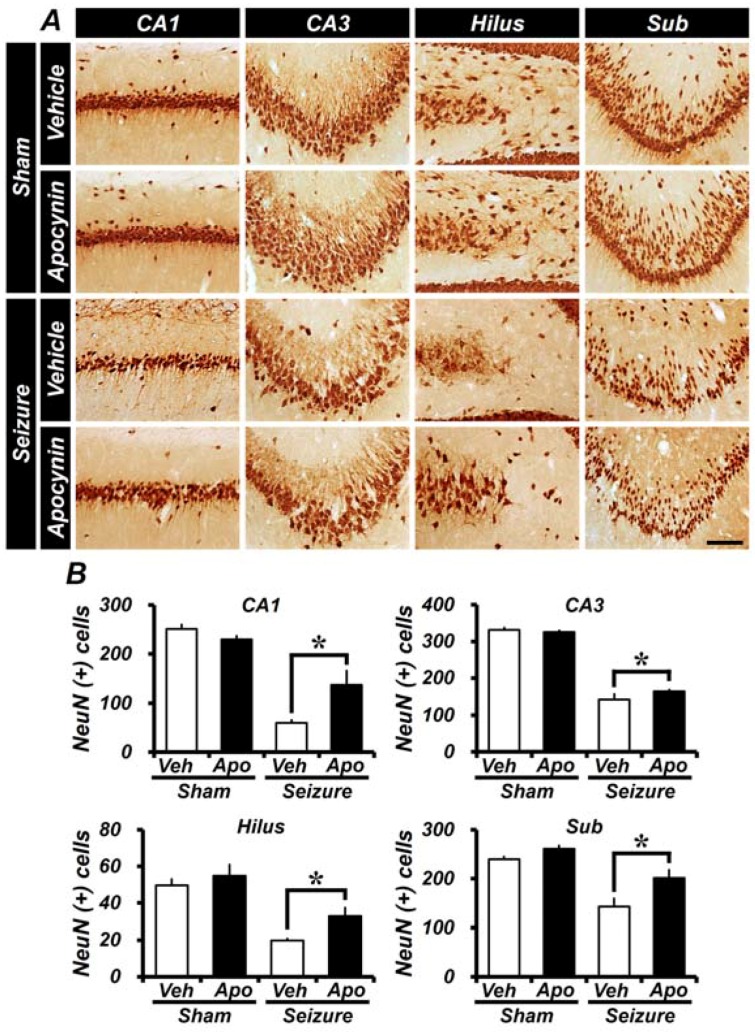
Apocynin treatment reduces neuronal cell loss induced by seizures. (**A**) Live neurons or mature neurons in the hippocampus were identified by NeuN immunohistochemical staining. Scale bar = 100 μm. (**B**) Bar graph showing the number of NeuN^+^ neurons in the hippocampus treated with apocynin for 4 weeks after seizures. Among the two groups receiving seizures, the apocynin-treated group had a greater number of neurons when NeuN^+^ cells were compared to the vehicle-treated group. Seizure-induced NeuN^+^ neuron loss was prevented by apocynin treatment in the hippocampus each region compared to the vehicle treatment. Data: Mean ± S.E.M., *n* = 5–8 from each group, * *p* < 0.05.

Neuronal cell death was assessed by immunohistochemical staining using NeuN, and the effect of apocynin on neuronal cell death was confirmed in hippocampal CA1, CA3, hilus, and subiculum areas. Four weeks after pilocarpine-induced seizures, NeuN^+^ neurons in each area of the hippocampus were found to have a reduced number of live neurons in both seizure-induced groups compared to the sham-vehicle group. The number of live neurons in the seizure-vehicle-treated group in CA1, CA3, the DG, and the subiculum was reduced compared to the sham-vehicle-treated group. However, in animals treated with apocynin, the number of NeuN positive cells was higher than that of the seizure-vehicle-treated group than the seizure group. These results show that apocynin treatment, even given 4 weeks after the induction of seizures, reduces neuronal damage that occurs in this setting (Figure 4).

### 2.5. Treatment of Apocynin for 4 Weeks after Seizures Reduces Oxidative Damage in the Hippocampus

**Figure 5:**
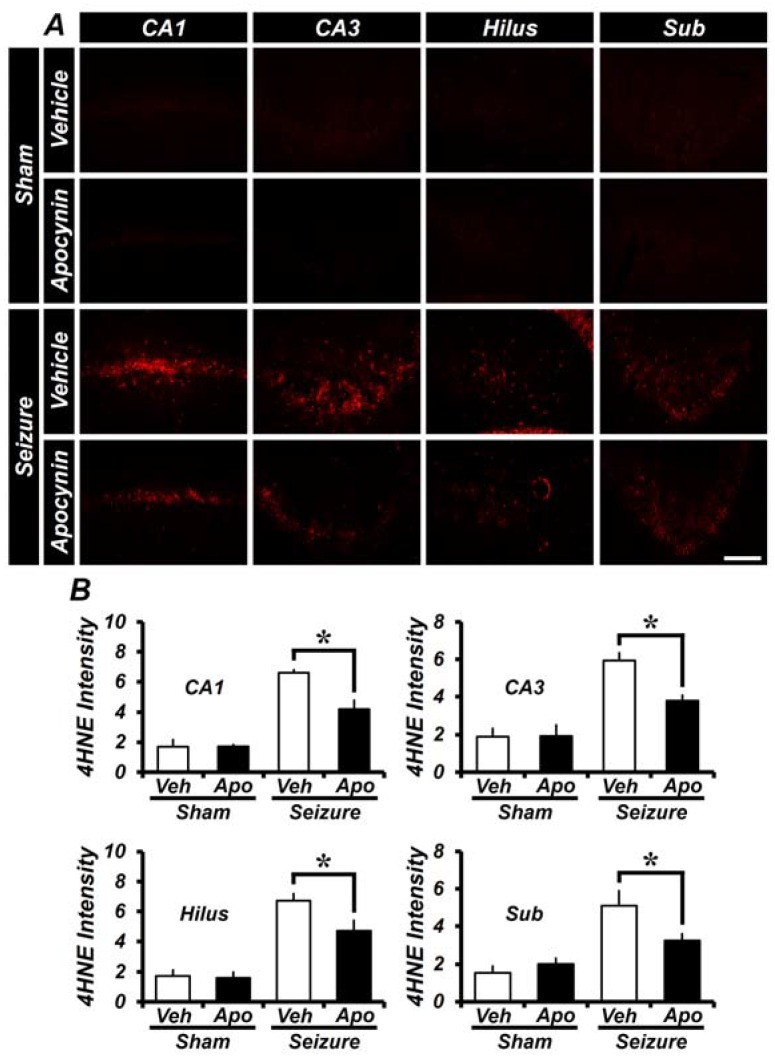
Apocynin treatment reduces 4HNE levels in the hippocampus at 4 weeks after seizures. (**A**) A microscopic image showing oxidative injury with 4HNE at each region of the hippocampus. The intensity of 4HNE was increased in the seizure group when compared to the sham-vehicle group. The 4HEN intensity was reduced in the seizure-apocynin treatment group when compared to the seizure-vehicle group. Scale bar = 100 μm. (**B**) The bar graph shows the quantification of the levels of 4HNE in each region of the hippocampus. Data: Mean ± S.E.M., *n* = 5–8 from each group, * *p* < 0.05.

A 4HNE (4-hydroxy-2-nonenal) antibody was used to evaluate the occurrence of oxidative damage in the hippocampus of the brain when apocynin was administered for 4 weeks after seizure. Figure 5B shows that the intensity of 4HNE in the seizure-vehicle group increased in the hippocampal region, when compared to the sham-vehicle group. The seizure apocynin-treated group had a lower 4HNE intensity than the vehicle-treated group. CA1, CA3, hilus and subiculum in the apocynin-treated group were reduced by 31 ± 8.7%, 36 ± 4.8%, 30 ± 8.1% and 40 ± 6.8%, compared to the vehicle-treatment group (Figure 5).

### 2.6. Apocynin Treatment Increases the Survival Rate of Newly Born Neurons and Newly Generated Neurons

**Figure 6:**
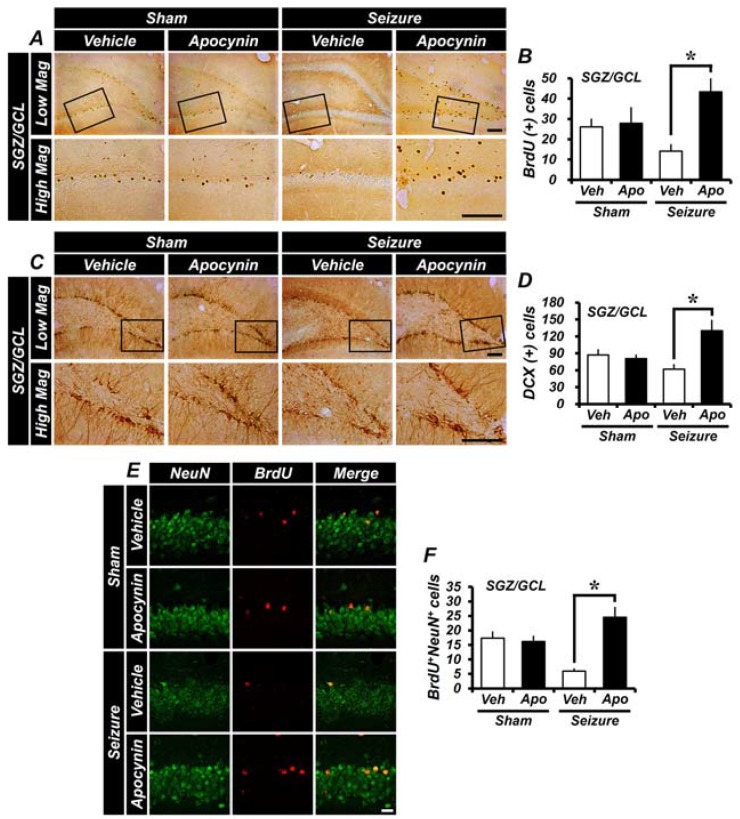
Apocynin treatment increases the survival rate of newly generated cells and neuroblast production in the SGZ of the DG. The survival rate of newly generated cells was assessed by BrdU staining. Long-term survival of newly proliferated cells was increased by apocynin treatment. Differentiation of newly proliferated cells into mature neurons was increased by apocynin treatment. (**A**) BrdU^+^ cells are shown in the SGZ of the DG. Four weeks after injecting BrdU in an adult (8–9 weeks old), BrdU-labeled cells can be seen in the granule cell layer. After 4 weeks of inducing seizures, the number of cells labeled with BrdU decreased. The apocynin-treated group had a decreased number of BrdU-labeled cells after seizures, but still greater than the vehicle-treated group. Boxed areas are higher magnification (bottom row). Scale bar = 100 μm. (**B**) The graph shows BrdU-labeled cells quantified in the hippocampus. The number of BrdU^+^ cells (survived for 4 weeks) was significantly higher in apocynin-treated rats than in vehicle-treated rats. (**C**) Immunohistochemical staining of this figure confirmed the presence of neuroblasts. In the seizure model, when the DCX^+^ cells were identified in the SGZ of the DG 4 weeks after apocynin treatment, the number of cells was significantly increased compared with the vehicle-treated group. Boxed areas are higher magnification (bottom row). Scale bar = 100 μm. (**D**) The graph is the mean value obtained from five individual sections and represents the number of DCX^+^ cells in the SGZ of the DG. (**E**) BrdU^+^NeuN^+^ cells are shown in the hippocampal DG region. BrdU^+^NeuN^+^ cells were identified in the granule cell layer at 4 weeks after BrdU injection. Scale bar = 100 μm. (**F**) The graph shows BrdU/NeuN labeled cells quantified in hippocampus. The number of BrdU^+^NeuN^+^ cells (survival for 4 weeks) was significantly higher than in the vehicle-treated group in the apocynin-treated group. Data: Mean ± S.E.M., *n* = 5–8 from each group, * *p* < 0.05. Low Mag, low magnification; High Mag, high magnification.

Cellular proliferation is increased in the early stages after a seizure, but survival of proliferated cells is not maintained 4 weeks post-insult [4,16]. The BrdU experiments at 1 and 4 weeks show that the proliferating cells, which were increased 1 week after seizures, were decreased after 4 weeks. BrdU staining was performed to confirm the survival of initially labeled BrdU^+^ cells after apocynin treatment for 4 weeks after seizures. After 4 weeks of seizure induction, it was confirmed that the number of BrdU-labeled cells at the early time of seizures decreased. Apocynin was injected for 4 weeks after seizures, and the survival count of the initially generated cells was found to be higher than in the vehicle-treated group. Furthermore, the survival of proliferating cells after a seizure was increased by continuous apocynin treatment more than the vehicle treatment (Figure 6A,B). Neuronal proliferation increased in the early stages after seizures. Analysis of neuroblasts 4 weeks after induction of seizures with pilocarpine showed the following results. Analysis of the number of neuroblasts revealed that the number of neuroblasts in the chronic interstitial hippocampus was significantly reduced. The density of newborn neurons in the SGZ was considerably greater after seizure-induced cell loss. At 4 weeks of pilocarpine-induced seizures, the neuroblast production in the vehicle-treated group was decreased compared to the initial vehicle-treated group. However, neuroblast production in the apocynin-treated group was higher than the vehicle-treated group (Figure 6C,D). BrdU and NeuN double staining was performed to determine whether the proliferating cells were differentiated into mature neurons. It was confirmed that the number of BrdU^+^NeuN^+^ cells labeled 4 weeks after seizures was decreased. Apocynin was injected for 4 weeks after seizures and the number of differentiated mature neurons among surviving cells was higher than in the vehicle group (Figure 6E,F).

## 3. Discussion

Epilepsy is known to occur after primary damage due to cerebral infarction, stroke, or traumatic brain injury. Epilepsy is one of the most common neurological disorders, and it is reported that approximately 50 million people worldwide suffer from this disease. Repeated epilepsy can result in damage to the temporal lobe, especially the hippocampus [32]. Studies on epilepsy with animal models and evaluation of human brain tissue have revealed the cause of neuronal death including partial regression of CA1 and CA3 pyramidal neurons, dentate gyrus (DG), and subiculum (Sub) cells in the hippocampus, but the exact causes and treatments are yet to be identified.

The effect of short-term apocynin administration before and after the onset of epilepsy has been previously studied [21,23,24]. However, the long-term post-treatment effect of apocynin has not been tested. Neuron nuclear antigen (NeuN), bromodeoxyuridine (BrdU), and doublecortin (DCX) staining was performed to evaluate whether apocynin administration has a neuroprotective effect and can increase the survival rate of newly generated neurons. When hippocampal neurons were injured in a pilocarpine-induced epilepsy model, cellular proliferation measured by BrdU labeling was dramatically increased in the subgranular zone (SGZ) of the DG. The number of neuroblasts also increased two to three days after epilepsy.

NADPH oxidase has also been reported to be involved in the neurodegenerative process of NADPH oxidase-dependent ROS overproduction in other brain regions, such as the cerebellum, that are involved in the transactivation of CAMKII [33]. It is known that epilepsy and oxidative stress are closely interrelated. It is also known that an increase of free radicals and oxidative stress after epilepsy can lead to neuronal cell death, because the brain is known to be more vulnerable to damage by excess oxygen and oxidative stress than other tissues [34,35]. Apocynin is known to reduce oxidative damage through the inhibition of NADPH oxidase activation. To confirm whether apocynin has neuroprotective effects, we performed NeuN staining 1 week or 4 weeks after seizures. At each time point, the apocynin-treated group, compared with the vehicle-treated group, showed a higher number of viable neurons in the hippocampus. These results indicate that apocynin therapy either for 1 week or for 4 weeks reduces neuronal death and increases the number of surviving neurons after seizures.

Oxidative damage is involved in promoting apoptosis and is thought to play an important role as a causative agent of degenerative neuropathy [36,37]. In this study, we found that the oxidative damage of neurons increased after one and four weeks of seizures. Administration of apocynin reduced oxidative damage in seizure-induced rats. This finding confirms and extends the idea that apocynin reduces oxidative stress and thus reduces the death of newly generated cells after seizures, as well as existing neurons.

Recently, it has been demonstrated that NADPH oxidase-mediated ROS generation can contribute to the transactivation of TRKs (Tropomyosin receptor kinases), thus improving cell proliferation and viability [38]. Oxidative stress leads to the death of existing neurons, but also of newly generated cells, after seizures [39]. Since apocynin is known to prevent neuronal death, the present study was performed to test whether apocynin also prevented the death of newly generated cells in pilocarpine-induced epilepsy models. Previous studies have reported that neuronal development or new-cell generation is continuously decreased in the SGZ of the adult rat hippocampus after a seizure [30,31,40,41,42]. In the present study, we also found that the number of newly generated cells after a seizure is lower than in sham-group animals, which means either the new cellular proliferation rate is lowered, or that the newly generated cells were damaged. Cellular proliferation in the hippocampus was observed at one week after the pilocarpine-induced seizure, but there was no difference between the control and apocynin-treated groups. Thus, the present study was undertaken to evaluate the effect of apocynin treatment on the survival rate of neurons and newly formed cells in the early stages of epilepsy. We found that the survival rate of BrdU-labeled cells, when evaluated 4 weeks after the seizure, was only 20% in the SGZ of the DG. However, at 4 weeks, the apocynin-treated group showed that the survival rate of BrdU-labeled cells was 40%. This result shows that apocynin treatment increases the survival rate of early-proliferated cells. The present study also shows that immediate administration of apocynin after seizures, for 1 week, was effective in decreasing neuronal cellular death, but administration of apocynin showed no beneficial effects on neuroblast generation. The number of neuroblasts after seizures was similar in both apocynin- and vehicle-treated rats one week after the seizure. However, we found that administration of apocynin for 4 weeks showed an increased number of neuroblasts in the hippocampus compared to the vehicle-treated group. It has been found that apocynin reduces oxidative injury and reduces apoptosis of newly generated neurons, leading to oxidative injury and to the death of newly generated cells, as well as existing neurons. This study shows that damage caused by oxidative injury after seizures was reduced by apocynin, thereby reducing the death of generated cells, as well as existing neurons, after seizures. This result demonstrates that inhibition of ROS production by apocynin increases neuroblast survival after seizure.

Two key results of the present study are as follows: (1) Apocynin increases the survival rate of newly formed cells; (2) apocynin increases the number of neuroblasts in the hippocampus after pilocarpine-induced seizures.

## 4. Materials and Methods

### 4.1. Ethics Statement

This study was performed in accordance with the guidelines of the Laboratory Animal Care and Use Guide issued by the National Institutes of Health (NIH). Animal studies are consistent with the requirements of the Hallym University Animal Care Committee (Protocol # Hallym 2016-70, approval date: 9 February 2016). Every effort, such as the use of isoflurane anesthesia, was made to minimize the pain of animals sacrificed.

### 4.2. Experimental Animals

In this study, 8-week-old Sprague-Dawley male rats (250–300 g, Daehanbiolink (DBL) Co., Chungcheongbuk-do, Korea) were used. Subjects were maintained at a constant room temperature (22 ± 2 °C) and humidity (55 ± 5%), one per cage, and the room lighting was automatically turned on and off at every 12 h (6:00 a.m. and 6:00 p.m.). The animals were divided into four groups for Week 1 (sham-vehicle *n* = 5; sham-apocynin *n* = 5; seizure-vehicle *n* = 6; seizure-apocynin *n* = 6) and Week 4 (sham-vehicle *n* = 5; sham-apocynin *n* = 5; seizure-vehicle *n* = 6; seizure-apocynin *n* = 8) groups.

### 4.3. Experimental Procedures

**Figure 7:**
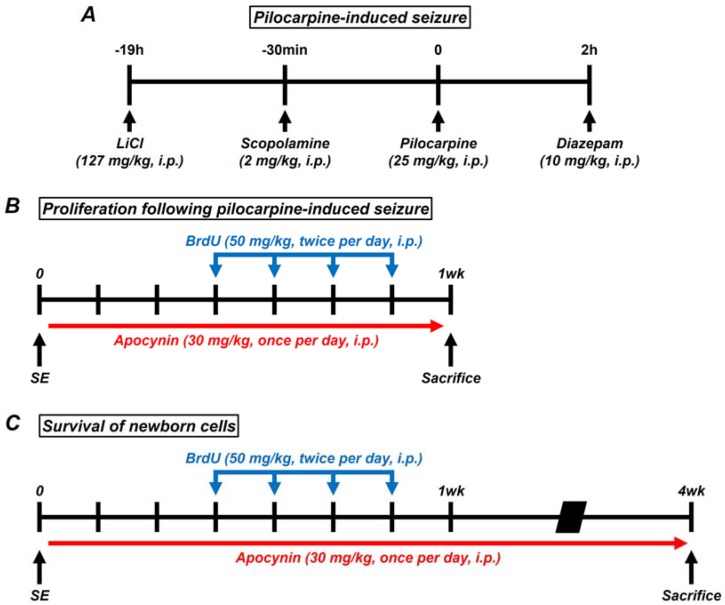
(**A**) Schematic of pilocarpine-induced seizure. Lithium chloride (LiCl 127 mg/kg, i.p.) was administered 19 h before induction of seizure by pilocarpine. Scopolamine (2 mg/kg, i.p.) was injected 30 min before administered pilocarpine. Pilocarpine was injected at 25 mg/kg, i.p. After a seizure with pilocarpine, diazepam (10 mg/kg, i.p.) was administered 120 min later to lower the seizure. (**B**,**C**) Schematic of key experiments performed in this study. Proliferation cells were confirmed in rats after pilocarpine-induced seizures. BrdU^+^ cells were quantified 1 or 4 weeks after the seizure. SE, status epilepticus.

Lithium chloride (LiCl, 127 mg/kg, i.p., Sigma-Aldrich Co., St. Louis, MO, USA) was administered 19 h before the injection of pilocarpine and induced epileptic seizures with pilocarpine (25 mg/kg, i.p., Sigma-Aldrich Co., St. Louis, MO, USA) [43]. Scopolamine (2 mg/kg, i.p., Sigma-Aldrich Co., St. Louis, MO, USA) was injected 30 min before the induction of seizures with pilocarpine. Seizures are generally known to occur 20–30 min after injection of pilocarpine [44]. Pilocarpine was administered only once at the day of the experiment in which the epilepsy is induced. Animals were housed one per cage for behavioral observations of individual seizures. According to the Racine stage, described by Racine, five symptoms are described when a seizure occurs (1. Mouth and facial movements; 2. Head nodding; 3. Forelimb clonus; 4. Rearing with forelimb clonus; and 5. Rearing and falling with forelimb clonus). We judged that a complete seizure has occurred as soon as the fifth symptom is observed [45]. The seizure was maintained after the seizure was complete, and diazepam (Valium, 10 mg/kg, i.p., Hoffman la Roche, Neuilly sur-Seine, Roche Co., Basel, Switzerland) was injected 2 h later. Diazepam (2 mg/kg, i.p.) was injected to stop residual seizure activity when recurrent severe seizures were observed after diazepam treatment [21,46] (Figure 7A). Apocynin and 5-bromo-2-deoxyuridine (BrdU) injections were divided into four groups of animals: Sham-dimethyl sulfoxide (DMSO) (Sigma-Aldrich Co., St. Louis, MO, USA, 0.01 mL/kg, pH 7–7.4, i.p.), sham-apocynin (Sigma-Aldrich Co., St. Louis, MO, USA, 30 mg/kg, i.p.), seizure-DMSO, and seizure-apocynin. Animals were injected with apocynin once a day for 1 week and 4 weeks after pilocarpine-induced seizures. Apocynin was injected 2 h after the seizure, and 2 injections 24 h after the seizure. After one week of seizures, BrdU (50 mg/kg, i.p., Sigma-Aldrich Co., St. Louis, MO, USA) was administered into intraperitoneal space twice a day, from 4 days before sacrifice, for 4 days to observe cell proliferation. In addition, to investigate the survival of the proliferated cells, animals were sacrificed 4 weeks after seizure (Figure 7B,C).

### 4.4. Brain Sample Preparation

All groups were sacrificed at 1 or 4 weeks after seizures. Animals were anesthetized with isoflurane and then perfused with 0.9% normal saline through the heart. Next, a brain sample was obtained by perfusion with 4% paraformaldehyde and fixed in the same paraformaldehyde fixative for 1 h. One hour later, the brain was immersed in 30% sucrose overnight. Usually, 1–2 days after a brain sample is taken, the sample sinks to the bottom of the sucrose solution. After the entire brain is frozen in the cryosection machine, the sample was cut into a 30-μm-thick cryostat at –20 to –23 °C. The brain sections were stored at −20 °C in a stock solution until histological evaluation.

### 4.5. Detection of Live Neurons

NeuN immunohistochemical staining to confirm live neuron was performed to evaluate whether a neuroprotective effect was obtained when apocynin was given after the pilocarpine-induced seizure. All immunostaining was performed on brain tissue sections (sliced at a 30 μm thickness with a cryosection machine), and each of the five sections were analyzed. Monoclonal antibody-NeuN antibody (diluted 1:500, Billerica, Millipore Co., Burlington, MA, USA) was used as the primary antibody and incubated at 4 °C. At the end of each step, each sample was washed three times with phosphate buffered saline (PBS) for 10 min. Brain sections were diluted 1:250 with secondary antibody biotinylated anti-mouse immunoglobulin G (IgG, Vector, Burlingame, CA, USA) in the same manner as the primary antisera. After 2 h of incubation at room temperature (RT), tissues were washed and incubated with the ABC compound (Vector, Burlingame, CA, USA). Tissues were stained with the 3,3-diaminobenzidine (DAB, Sigma-Aldrich Co., St. Louis, MO, USA) color development method, and the sections were plated on slides [47].

### 4.6. Detection of BrdU Labeling

To determine the amount of newly generated cells and the effect of apocynin on the long-term survival and differentiation of these cells, BrdU was injected into the experimental group at 12 h intervals every day for 4 days. For immunoperoxidase detection of cells with BrdU incorporated, the sections were incubated with 0.3% hydrogen peroxide in methanol for 15 min. The sections were incubated in 2 N HCl for 90 min to denature the DNA and prepare for BrdU immunostaining, and then washed twice with 0.1 M sodium borate buffer for 10 min to neutralize the acid. At the end of each step, each sample was washed three times with phosphate buffered saline (PBS) for 10 min. In a solution containing 1% normal chicken serum and Triton X-100, a monoclonal antibody-BrdU antibody (diluted 1:150, Roche Co., Basel, Switzerland) was used as the primary antibody and incubated at room temperature for 1 h. After attaching the primary antibody, the sections were incubated with secondary antibody (diluted 1:250 anti-mouse IgG) for 2 h, at RT. Tissues were subjected to a RT reaction in a biotin-peroxidase complex (ABC, Vector Lab., Burlingame, CA, USA) for 2 h, and then visualized using 3,3′-Diaminobenzidine (DAB, Sigma-Aldrich Co., St. Louis, MO, USA). Double immunofluorescence was used to confirm that the newly generated cells were differentiated into mature neurons. The sections were incubated with primary antibody for 2 h, at RT, in mouse anti-NeuN (diluted 1:500, Billerica, Millipore Co., MA, USA) and rat anti-BrdU (diluted 1:150, Roche Co., Basel, Switzerland). Two hours later, the sections were washed three times with PBS and diluted with Alexa Fluor 488 donkey anti mouse IgG (NeuN) and Alexa Fluor 594 donkey anti-rat IgG (BrdU) secondary antibody (diluted 1:250, Invitrogen, Grand Island, NY, USA) incubated for 2 h, at RT. BrdU and NeuN (Alexa 594 and 488) were used in the scanning mode using a Zeiss LSM 710 confocal imaging system (Carl Zeiss, Oberkochen, Germany) to detect fluorescent signals.

### 4.7. Doublecortin (DCX) Immunohistochemistry

To detect neuroblasts, brain slices were immunostained with guinea pig anti-doublecortin (DCX) antibody (1:1000, Santa Cruz Biotechnology, Santa Cruz, CA, USA) [48]. Brain sections were incubated overnight at 4 °C with primary DCX antibody. The sections were incubated with secondary antibody (diluted 1:250 guinea pig IgG) for 2 h, at RT. Images of neuroblast were captured using an Olympus IX70 inverted microscope (Olympus Co., Tokyo and Shinjuku, Japan). The experimenter counted the number of neuroblasts in the subgranular zone (the SGZ in the DG) of both hippocampal lobes. The number of neruoblasts in the SGZ was quantified by the experimenter.

### 4.8. 4HNE (4-Hydroxy-2-Nonenal) Immunohistochemistry

Detection of oxidative damage in the hippocampal region of brain by 4HNE (4-hydroxy-2-nonenal) staining was evaluated by confirming lipid peroxidation products. 4HNE (Alpha Diagnostic International Inc., San Antonio, TX, USA) antibody immunostaining was performed as described above. The tissues were incubated overnight at 4 °C in PBS containing 0.3% Triton X-100 in a polyclonal rabbit anti-HNE antiserum compound (diluted 1:500, Alpha Diagnostic International Inc., San Antonio, TX, USA). After tissue incubation, brain tissue was incubated with Alexa Fluor 594 conjugated goat anti-rabbit IgG secondary antibody (Invitrogen, Grand Island, NY, USA) at a dilution of 1:250 for 2 h, at RT. After secondary staining, the sections were washed three times for 10 min with PBS and plated on gelatin-coated slides. The stained tissue was observed for fluorescence using an Axioscope microscope (Carl Zeiss, Munchen Hallbergmoos, Germany) (Alexa 594) and the 4HNE intensity was quantified using image J (Laboratory for Optical and Computational Instrumentation (LOCI), University of Wisconsin, Madison, WI, USA).

### 4.9. Data Analysis

We compared each test group using repeated measures versus deviation analysis (ANOVA) and a Bonferroni post-test. Data were expressed as mean ± standard error of the mean (S.E.M.) and were considered significant when *p* < 0.05.
